# Impact of community-driven interventions on dietary and physical activity outcomes among a cohort of adults in a rural Appalachian county in Eastern Kentucky, 2019–2022

**DOI:** 10.3389/fpubh.2023.1142478

**Published:** 2023-04-14

**Authors:** Heather Norman-Burgdolf, Emily DeWitt, Rachel Gillespie, Kathryn M. Cardarelli, Stacey Slone, Alison Gustafson

**Affiliations:** ^1^Department of Dietetics and Human Nutrition, College of Agriculture, Food and Environment, University of Kentucky, Lexington, KY, United States; ^2^Department of Health, Behavior & Society, College of Public Health, University of Kentucky, Lexington, KY, United States; ^3^Dr. Bing Zhang Department of Statistics, College of Arts & Sciences, University of Kentucky, Lexington, KY, United States

**Keywords:** cooperative extension, rural, prospective cohort study, diet, physical activity, PSE

## Abstract

Several environmental level factors exacerbate poor health outcomes in rural populations in the United States, such as lack of access to healthy food and locations to be physically active, which support healthy choices at the individual level. Thus, utilizing innovative place-based approaches in rural locations is essential to improve health outcomes. Leveraging community assets, like Cooperative Extension, is a novel strategy for implementing community-driven interventions. This prospective cohort study (n = 152), recruited in 2019 and surveyed again in 2020 and 2021, examined individual level changes in diet and physical activity in one rural Appalachian county. During this time, multiple community-driven interventions were implemented alongside Cooperative Extension and several community partners. Across the three-year study, the cohort indicated increases in other vegetables and water and reductions in fruits and legumes. There were also reductions in less healthy items such as French fries and sugar-sweetened beverages. The cohort also reported being less likely to engage in physical activity. Our findings suggest that key community-driven programs may have indirect effects on dietary and physical activity choices over time. Outcomes from this study are relevant for public health practitioners and community organizations working within rural Appalachian communities to address health-related behaviors.

## 1. Introduction

Rural Appalachian residents have the highest cancer incidence rates and cancer mortality rates relative to other rural and urban areas (1, 2). In addition, rural residents experience higher rates of type 2 diabetes relative to their urban counterparts (3). Specifically, Kentucky ranks 4^th^ highest in the United States (U.S.) for mortality rate from diabetes, and in the Appalachian community under study, the adult prevalence of diagnosed diabetes is 17% compared to 12% in non-Appalachia Kentucky (4).^1^ Lastly, rural residents experience higher rates of food insecurity compared to their urban counterparts (5, 6). Recent studies have indicated that Kentucky has a food insecurity rate of 17% compared to the national average of 15% (7), while 22% of adults report being food insecure in Eastern Appalachian Kentucky (7).

Research to date has shown that rural communities face greater barriers to healthy eating, specifically low intake of fruits, vegetables, and high consumption of sugar-sweetened beverages, which contribute to higher rates of obesity (8), type 2 diabetes, and certain cancers (8–13). The culmination of poor access to healthy food options, resource poor neighborhoods, and a systemic high rate of poverty all perpetuate poor health outcomes among underserved Appalachian populations. Nutrition interventions and community-based programs may help to improve dietary intake. However, these efforts alone are not sufficient. In addition to the food environment, addressing the built environment for being physically active is of equal importance to support overall health and wellbeing.

Active living initiatives in urban environments have improved sidewalks, crosswalks, and bike lanes creating opportunities for active daily routines (14). However, rural areas are typically more dispersed and may lack a downtown setting where work, play, and recreation opportunities are concentrated within a connected walkable environment. Apart from dependence on automobiles for travel behavior, regular transit services and alternative travel modes face a range of economic and environmental challenges and limitations for rural residents (15). In addition, many rural residents may work beyond their county of residence, spending considerable time commuting and traveling for employment, errands, and other activities. Despite the environmental challenges of rural communities, walking trails are accessible and have been found to encourage physical activity (16). A current intervention in six rural communities has indicated that promotional efforts are needed for trail use (17, 18). However, there remains a large gap in the research as it relates to assessing infrastructure changes on the same residents over time in a rural community.

Considering the challenges and barriers rural communities face to promote and sustain health improvement, initiatives and interventions must consider community-driven and community-based approaches to improve health outcomes through nutrition and physical activity (19–21). This provides a localized mechanism that accounts for the culture and existing resources within a community that can be leveraged to mitigate public health challenges. Further, this approach ensures that newly developed efforts are acceptable, complementary, and not competing or conflicting with existing programs and initiatives in the community. However, public health approaches should recognize the influence that place-based challenges have on individual behaviors and choices. Research to date has found that utilizing several layers of influence on diet and physical activity outcomes produces greater effects rather than one individual or environmental construct (22). Previous studies have demonstrated the effectiveness of using community-based interventions in addressing health disparities and have called on non-traditional partners and community-based participatory approaches within rural settings to address public health issues through policy, systems, and environmental (PSE) work (23–25).

One important partner often found within rural communities is the Cooperative Extension Service (Extension). Extension is operated in partnership with federal, state, and local governments to increase access to agriculture, health, and community resources in or near all U.S. counties. Extension is uniquely situated in that it provides rural communities a direct connection to a land-grant university and maintains high levels of community trust and engagement. Extension is charged with providing evidence-based information to communities to address health issues at the local level. This is accomplished through community-level projects and programs with existing partners and coalitions *via* direct education related to food safety, nutrition education, and other topics related to family health.

Funding agencies are increasingly looking to Extension to address local needs through community-level work. Examples include the Robert Wood Johnson Well Connected Communities and the Center for Disease Control and Prevention’s (CDC) High Obesity Program (HOP), with overarching goals of reducing rural obesity prevalence and chronic disease risk (26–28).^2^ Overall, public health agencies and organizations are beginning to recognize the power of non-traditional partners like Extension to address broader health issues through their direct engagement with communities and the capacity to implement PSE work (29, 30). These collaborative efforts may positively impact rural communities’ health if tailored PSE strategies can be done in these settings.

While many studies have employed community-driven approaches to promote positive behavior change and improve health outcomes, there are few that intentionally utilize Extension to target both individual and environmental determinants of health. The purpose of this study is to determine the impact of community-driven evidence-based programmatic initiatives and PSE changes facilitated by Extension over a three-year period on 1) fruit and vegetable intakes, 2) beverage intakes, and 3) physical activity engagement among adult residents in a rural Appalachian county.

## 2. Methods

### 2.1. Study design

The study design is a prospective cohort among adults residing in Martin County, Kentucky, over the past three years (2019–2022). This study is part of the multi-year CDC HOP project, which provides cooperative agreements with land grant universities and Extension, in partnership with other community-based organizations, to implement PSE strategies to reduce or prevent adult obesity (31).^3^ The University of Kentucky Institutional Review Board approved all materials and procedures for this study (protocol #40895).

### 2.2. Setting

Martin County, Kentucky, shares a border with West Virginia and is located in the Central Appalachian region. This county has a population of 11,140, and exhibits an obesity prevalence of 45% compared to the state and national average of 40 and 42%, respectively (32, 33).^4^^,^^5^ This community experiences persistent poverty, with a median household income of $29,387 USD (32). Currently, an estimated 41% of individuals live in poverty and 24% of the community residents participate in the Supplemental Nutrition Assistance Program (SNAP) (34).^6^ Disability rates are high among county residents, with 23% of adults under the age of 65 living with a disability (32). Life expectancy in this county is approximately 5 years below the national average (77.3 years) (35).^7^

### 2.3. Cohort recruitment and retention

The original cohort was recruited in Fall 2019 with n = 152. Details pertaining to the complete methodology have been previously reported (36). Inclusion criteria were consistent across all three time points. To compile time points following baseline in 2019, efforts to reach, re-engage, and collect survey responses *via* telephone were conducted in Fall 2020 through Winter 2021 to establish time point two (n = 74). Additionally, these findings, comparisons, recruitment strategies, and methodology have been previously reported (37). At time point three, the study team re-engaged participants and the final sample was n = 93. Postcards were mailed to all cohort participants in April 2022 inviting them to schedule their survey for time point three, and again in May 2022 to any remaining participants that had not been reached out or scheduled for a survey. If necessary, three phone call attempts were then made after the initial postcard mailing by study personnel to re-engage cohort participants for the final time point. Time point three data collection occurred late spring and early summer of 2022.

**Figure 1:**
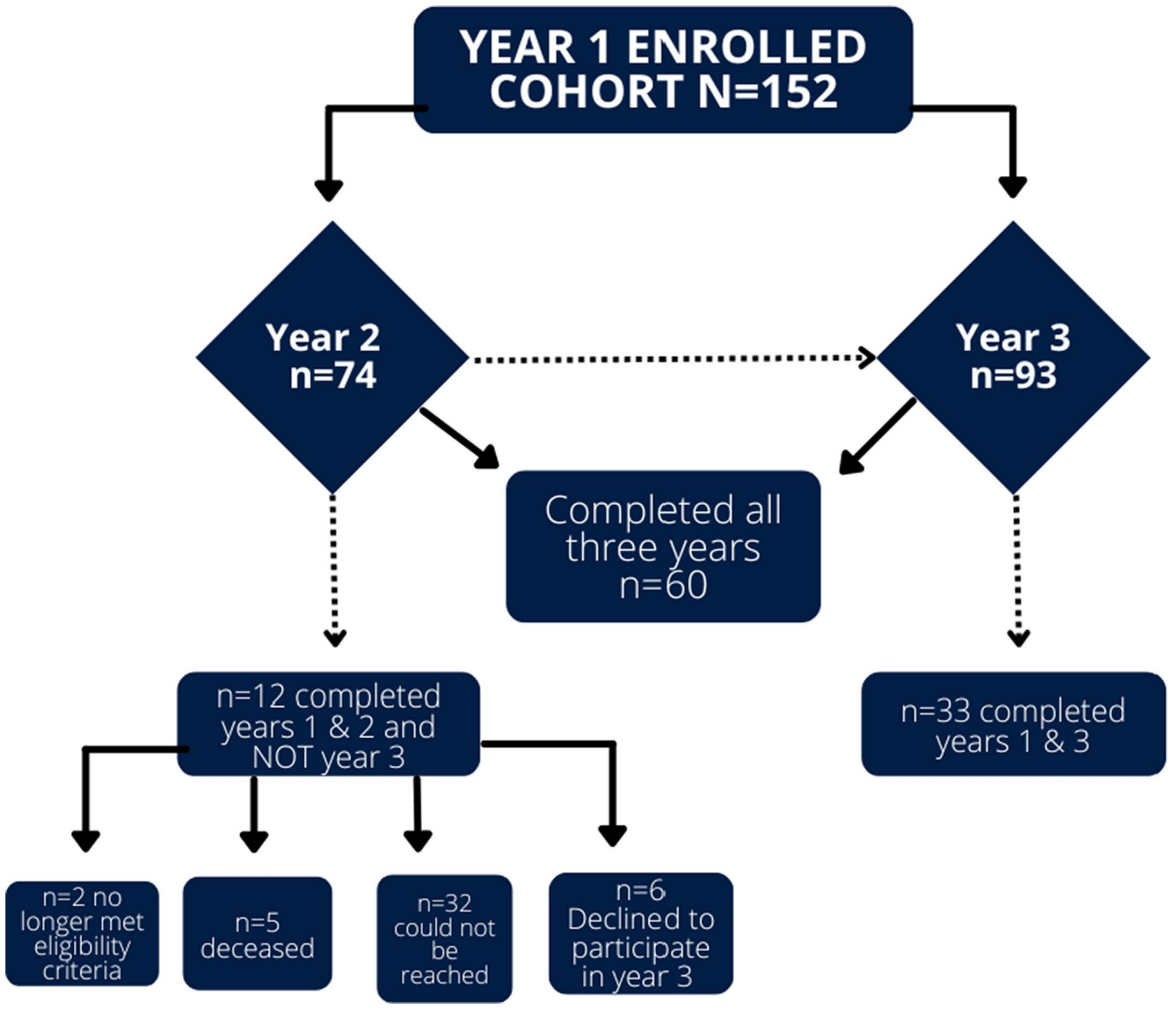
Retention and reporting for cohort study participants across the three separate time points.

Figure 1 outlines the response and completion rate from each of the attempts. A total of n = 93 surveys were ultimately completed for time point three; n = 60 completed all three time points, n = 33 completed surveys at time point one and time point three, but not time point two; and n = 12 completed surveys at time points one and two but not time point three. Several factors impacted retention over the three-year period (Figure 1). However, the greatest challenge when re-engaging participants was communication difficulties. These included numerous non-working telephone numbers and poor reception and connectivity common within the Appalachian region. Sample size at time point two may have also been impacted by the COVID-19 pandemic with data collection occurring during the peak of new cases in Kentucky. Of those within the original cohort (n = 152) that did not complete a survey at time point three, n = 6 declined to participate, n = 32 could not be reached after the multiple attempts, n = 2 no longer lived in Martin County, and n = 5 were deceased. Retention from baseline to time point three was 61.2%. No significant differences in baseline attributes were found between responders and non-responders at time point three (data available upon request).

### 2.4. Summary of interventions

Since 2019, several community-driven interventions guided by Extension at the county-level were implemented to address healthy food access and physical activity opportunities. The focus of these interventions was on PSE changes that made healthy choices easier and more feasible within the community. These community-driven interventions were informed by annual focus groups with local residents and stakeholders and completed in partnership with the local Wellness Coalition, community organizations, and Extension (38–40). Some interventions were specifically selected due to their demonstrated effectiveness of eliciting behavior change in similar communities (41–45).^8^ Table 1 provides a summary of community-based interventions implemented with relevant community partners in our setting from 2019–2022. Food access interventions aimed to enhance the existing food system to increase access to healthier foods. Physical activity interventions aimed to connect sidewalks, walking paths, schools, sites, parks, or recreation centers through master plans and land use interventions.

**Table 1:** Community-based interventions implemented within the specified county during the study period (2019–2022).

Intervention	Behavior Change Goal	Involved Community Partners
Food Systems/Food Access		
Make improvements to state and local programs/systems		
Increase capacity of local food pantries	Increase consumption of fruits and vegetables	Three Martin County food pantries
Establish healthy nutrition standards in key institutions		
Faithful Families Programming	Increase consumption of fruits and vegetables; increase water consumption	Two local faith-based organizations
Work with food vendors, distributors, and producers to enhance healthier food procurement and sales		
Pop Club Programming	Increase consumption of fruits and vegetables	Martin County Extension; Martin County Farmers’ Market
Smart Snacks Marketing Campaign	Increase consumption of nutritious snack choices; increase water consumption	Five local gas stations
Community garden	Increase consumption of fruits and vegetables	One local faith-based organization
Hydroponic units	Increase consumption of fruits and vegetables	Two local schools (one elementary, one middle); Senior citizens center
Physical Activity		
Improved pedestrian, bicycle, or transit transportation systems that are combined with new or improved land use or environmental design		
StoryWalk® installation on two walking trails	Increased engagement in physical activity	Martin County Extension; Martin County elected officials, Martin County Public Library
Park revitalization efforts (e.g., tennis court nets)	Increased engagement in physical activity	Martin County elected officials; Warfield Park Board; Martin County Public Library
Community walk/run events (e.g., Turkey Trot, 2 K Summer Dash)	Increased engagement in physical activity	Martin County Extension; Martin County Public Library

Implementation of food access and physical activity interventions was accomplished in collaboration with a variety of community partners. Most of these community partners were represented in the local Wellness Coalition that guided the development and implementation of interventions. This local coalition served as a community advisory board and was comprised of local health advocates, concerned citizens, and members and staff of the following community organizations: local government, faith organizations, tourism board, school food service, senior citizens center, public library, non-profits, food pantries, local park boards, and afterschool programs. Extension identified relevant community partners to participate in the Wellness Coalition and provided a physical meeting space for the advisory board. The Wellness Coalition was maintained throughout the study period by quarterly meetings and regular correspondence through email and newsletters.

### 2.5. Survey administration across three time points

The survey instrument was developed to align with required outcomes for the HOP cooperative agreement as outlined per the funding agency. Key outcomes of interest included improved dietary habits, including fruit and vegetable consumption, and an increase in physical activity. The same survey was administered across all three time points. Each survey included questions from validated instruments including the National Cancer Institute Fruit and Vegetable Screener (ref),^9^ the Beverage Intake Questionnaire (BEVQ-15) (ref), and the Global Physical Activity Questionnaire (GPAQ) (ref).

Time point one surveys were administered in person and time points two and three were collected *via* telephone. All surveys were administered by trained study personnel. Each survey took approximately 45 min to complete, and all participants completing each time point received a $40 USD gift card for their time. The survey collected demographic characteristics and included questions related to food and beverage intake and physical activity engagement. Demographic characteristics included age (in years), self-identified gender, highest attained education or vocational training level, race and ethnicity, annual household income, and participation in local and federal nutrition assistance programs.

### 2.6. Primary and secondary study outcomes

#### 2.6.1. Fruit and vegetable intakes

Fruit and vegetable intakes were primary outcomes. To measure dietary intake patterns, the National Cancer Institute’s Fruit and Vegetable Intake Screener was used (46, 47) (see text footnote 9). Participants were asked about their usual intake of a variety of fruits and vegetables over the last month, including how frequently they consumed the items and how much they consumed each time (i.e., “Over the last month, how often did you eat lettuce salad (with or without other vegetables)?” and “Each time you ate lettuce salad, how much did you usually eat?”). Items assessed include fruit, 100% fruit juice, lettuce salad, French fries or fried potatoes, other white potatoes, cooked dried beans, other vegetables, tomato sauce, vegetable soup, and mixtures that included vegetables. Other vegetables include all vegetables in any form other than white potatoes, fries, legumes, or lettuce salads.

#### 2.6.2. Beverage intakes

Beverage intake was a secondary outcome and was captured using the validated BEVQ-15 (48, 49). Participants were asked about their beverage choices over the last month, including how frequently they consumed the beverages and how much they consumed each time. Items assessed include water, 100% fruit juice, sweetened juices, variety of milks, soft drinks, energy drinks, diet soft drinks and other artificially sweetened drinks, sweetened tea, black coffee or tea, coffee or tea with cream and/or sugar, and any alcohol. Validated equations were used to summarize daily beverage intakes, reported in calories and grams.

#### 2.6.3. Physical activity engagement

Physical activity was a secondary outcome and captured using the validated GPAQ (50). Participants were asked about their sedentary behavior and engagement in moderate-intensity and vigorous-intensity physical activity within a typical week recreationally, related to their work, and their travel to and from places. Frequency was assessed for participants with an affirmative response to questions by providing the number of days per week they engaged in the activity.

### 2.7. Data entry and analysis

Survey responses were recorded directly into the REDCap database (Vanderbilt University, Nashville, TN, USA) to be analyzed across and between time points. Collected data was exported and analyzed using SAS 9.4 (SAS Institute, Cary, NC, USA). Demographic variables were summarized using median and ranges for age and counts and percentages for all categorical measures. Differences between responders and non-responders were tested using Wilcoxon rank sum (age) and chi-square and Fishers Exact as appropriate. Dietary and beverage intake measures were tested using repeated measures linear mixed models to assess change across time points. An autoregressive correlation structure was used to account for the intrasubject correlation. Least-square means are reported at each time point. A composite exercise variable was created to measure any reported intense activity whether vigorous or moderate. To assess the changes in physical activity engagement, a generalized mixed model was used to model the dichotomous outcomes. The autoregressive correlation structure was also used for these models. Odds ratios with 95% confidence intervals are presented comparing the time points. For the changes in activity, responses of sometimes/often and usually/always were collapsed and compared against never/rarely responses.

## 3. Results

### 3.1. Study sample

Demographic characteristics of the cohort can be found in Table 2. Demographic characteristics for all Martin County residents as well as the United States are provided for comparison. No differences in attributes were observed between any of the years within the study. The sample was primarily comprised of white females with a high school degree or higher. Most participants reported an annual household income of less than $20,000 USD (n = 90, 60.4%), with over one-third participating in SNAP (n = 60, 39.5%). Only 4% (n = 6) and 13.8% (n = 21) of the cohort participated in the Women, Infant, and Children Special Supplemental Nutrition Program and the Senior Farmers’ Market Nutrition Program, respectively.

**Table 2:** Sociodemographic characteristics among the sampled cohort, compared to Martin County, KY, and the United States, 2019–2022.

Demographic Characteristic	Cohort *n* = 152% (n)	Martin County^1^%	United States^1^%
**Age (median (range), in years)**	56.0 (22–84)	40.3	38.8
**Gender**^**2**^			
Male	34.9 (53)	55.4	49.5
Female	65.1 (99)	44.6	50.5
**Race**			
White	98.7 (150)	91.3	75.8
Non-white	1.3 (2)	8.7	24.2
**Education**			
Less than high school	43.4 (66)	26.3	11.1
High school graduate or higher	56.6 (86)	73.7	88.9
**Household Income (USD)**			
< $20,000	60.4 (90)	$29,387 (median)	$69,717 (median)
≥ $20,000	39.6 (59)		
**SNAP Participation**			
Yes	39.5 (60)	24.0	11.4
No	60.5 (92)	76.0	89.6

USD = United States Dollar, SNAP = Supplemental Nutrition Assistance Program; ^1^ Analogous data from the U.S. Census Bureau Small Area Income Poverty Estimates (https://www.census.gov/programs-surveys/saipe.html); ^2^Male and female were the only genders selected by participants on the survey instrument.

### 3.2. Primary outcome: Fruit and vegetable intakes

The primary outcomes of interest for dietary intake indicated a significant increase in servings of other vegetables (*p* = 0.0029) with a decrease in fruit (*p* = 0.0338) and legume (*p* = 0.0286) consumption. However, there were also significant reductions in less healthy items, including French fries (*p* = 0.0334) during the same time frame. Dietary changes related to various fruits and vegetables among cohort participants can be found in Table 3.

**Table 3:** Dietary changes related to various fruits and vegetables among cohort participants, 2019–2022.

	Year 1 Mean (SE)	Year 2 Mean (SE)	Year 3 Mean (SE)	Overall value of p	ICC
Other Vegetables	0.61 (0.08)	0.93 (0.11)^+^	1.00 (0.10)^+^	0.0029	0.19
French Fry	0.73 (0.09)	0.65 (0.12)	0.40 (0.11)^*@^	0.0334	0.17
Fruit Servings	1.02 (0.10)	0.73 (0.12)^*^	0.76 (0.12)^*^	0.0338	0.32
Legumes	0.39 (0.04)	0.31 (0.05)	0.24 (0.05)^+^	0.0286	0.08
Juice Servings	0.42 (0.06)	0.31 (0.07)	0.42 (0.07)	0.2292	0.37
Fruit/Vegetable Servings	4.57 (0.31)	3.96 (0.38)	3.97 (0.38)	0.1846	0.26
Salad Servings	0.38 (0.07)	0.28 (0.08)	0.40 (0.08)	0.2942	0.56
White Potato	0.47 (0.04)	0.42 (0.05)	0.40 (0.05)	0.4018	0.22
Tomato Sauce	0.22 (0.04)	0.17 (0.05)	0.11 (0.05)	0.1441	0.07
Vegetable Soup	0.34 (0.07)	0.18 (0.10)	0.22 (0.09)	0.3366	0.03

SE = standard error, ICC = intraclass correlation; ^*^pairwise value of p vs Year 1 < 0.05, ^+^pairwise value of p vs Year 1 < 0.01, ^#^pairwise value of p vs Year 1 ≤ 0.001; ^@^ pairwise value of p vs Year 2 < 0.05, ^pairwise value of p vs Year 2 < 0.01.

### 3.3. Secondary outcome: Beverage intake

Overall, the sample consumed fewer total beverage calories from Year 1 to Year 3 (*p* = 0.0002). Similarly, participants self-reported significantly fewer sugar-sweetened beverage (SSB) calories (*p* = 0.0007) and milk calories (*p* = 0.0001) at the end of the study compared to Year 1. Water intake appeared to increase but the apparent trend did not reach statistical significance. Dietary changes related to beverages among cohort participants can be found in Table 4.

**Table 4:** Dietary changes related to beverages among cohort participants, 2019–2022.

	Year 1 Mean (SE)	Year 2 Mean (SE)	Year 3 Mean (SE)	Overall value of p	ICC
Water (grams)	861 (47)	966 (59)	962 (58)	0.1289	0.65
Total Bev Calories	481 (30)	398 (38)^*^	308 (37)^# @^	0.0002	0.43
Total Bev (grams)	2,472 (87)	2,264 (110)^*^	2060 (107)^#^	0.0029	0.39
SSB Calories	216 (17)	233 (22)	144 (21)^+ §^	0.0007	0.53
SSB (grams)	663 (46)	670 (59)	447 (57)^#§^	0.0006	0.57
Milk Calories	186 (16)	118 (20)^#^	103 (20)^#^	0.0001	0.59
Milk (grams)	305 (26)	197 (33)^#^	177 (32)^#^	0.0003	0.60
Alcohol Calories	5.8 (2.4)	3.9 (3.3)	4.3 (3.0)	0.8730	−0.02
Alcohol (grams)	10.4 (3.8)	0.8 (4.9)	2.9 (4.7)	0.2302	−0.01

SE = standard error, ICC = intraclass correlation, Bev = beverage, SSB = sugar-sweetened beverages; *pairwise value of p vs Year 1 < 0.05, +pairwise value of p vs Year 1 < 0.01, #pairwise value of p vs Year 1 < 0.001; @pairwise value of p vs Year 2 < 0.05, ^pairwise value of p vs Year 2 < 0.01, §pairwise value of p vs Year 2 < 0.001.

### 3.4. Secondary outcome: Physical activity engagement

**Figure 2:**
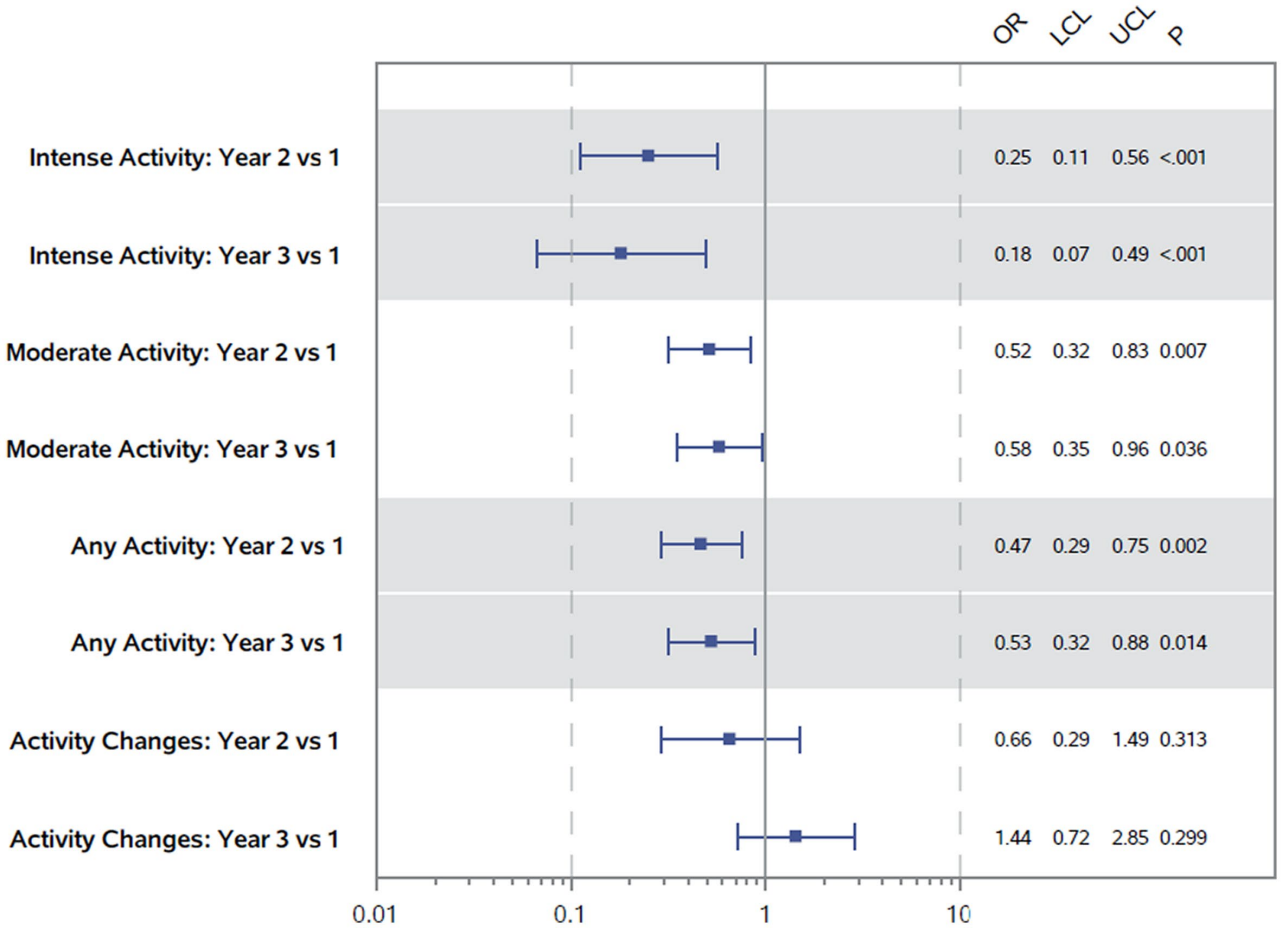
Physical activity levels among cohort participants, 2019–2022.

When comparing all time points, the cohort was less likely to engage in vigorous intense activity (*p* = 0.0004), moderate intense activity (*p* = 0.0171), or any level of activity (*p* = 0.0046) at the end of the study. There were no statistically significant differences for intensity of activity or activity levels between Years 2 and 3 (excluded from table). Figure 2 provides year-to-year comparisons for activity levels demonstrating that activity changes were not significant across the study period (*p* = 0.1818).

## 4. Discussion

This study utilized several community-led interventions in collaboration with Cooperative Extension and local partners that comprised the community advisory board. The programming efforts largely focused on built environment changes as a more upstream approach to improve dietary intake and physical activity. Although we cannot make a direct causal association between these programs and direct effects, our study can highlight how exposure to programming over time may have indirectly affected dietary intake and physical activity choices among adults in this rural setting.

The community-driven initiatives addressing dietary choices in this study aligned with previous research in Appalachia in which residents identified faith-based organizations, schools, and gardening as appropriate strategies to improve dietary choices (51). Our study utilized schools, faith-based organizations, non-profits, and local food retailers as key partners and locations for improving access to healthy food options. However, over time our results point to a complex relationship between community-driven built environment changes and subsequent changes in diet and physical activity. Shifts in vegetable consumption were observed, with fewer French fries and more diverse vegetables being consumed among the cohort over the three time periods. While these are promising findings regarding vegetables, there was a significant decrease in fruit consumption across all time points. The discrepancy in intake may be largely attributed to the fact that vegetables can be procured locally through home gardening, food pantries, and other food assistance programs. However, in our experience, fruit is not easily grown in the region and is primarily available in larger grocery stores and supermarkets which are less convenient (52–54). During this study time frame, there was a closure of a grocery store in the county seat which impacted where residents shopped for food (37). Although programming efforts aimed to target improved access, there were equal and opposite opposing forces limiting healthy options in the community.

An important finding was a significant reduction in the amount of SSBs consumed among the cohort during the study period. We have previously reported on the key role that gas stations play in purchasing habits in this community and how non-soda SSBs are a significant source of added sugar in the diet of adults in this community (55, 56). As a result, marketing strategies were deployed within gas stations to promote healthier beverage choices and nutritious snacks within this study. Although substantial changes in beverage purchases were not observed cross-sectionally (55), the cohort study allowed us to capture differences in consumption patterns over time. Although SSB consumption in the sample decreased, there were no significant changes in water consumption. However, this was not surprising to study personnel, as barriers to accessing water are prominent in this community. In addition to a poor food environment, Martin County has experienced a water crisis for more than two decades where county residents are not provided reliable or safe drinking water (57, 58). Unreliable access to safe drinking water, coupled with limited financial resources, makes it challenging for residents of this community to prioritize water consumption. It is evident that multiple, large-scale strategies are required to result in subtle dietary changes. Food access, existing infrastructure, and availability of items should be included in interventions seeking to support positive health behavior, especially in rural Appalachian communities.

Other health behaviors were examined across the study time frame. Notably, all forms of physical activity decreased, and participants were less likely to engage in any level of physical activity at the end of the study. While we had promising coalition and community support for initiatives (i.e., StoryWalk®), we were unable to make substantial changes to built environment infrastructure such as sidewalks, crosswalks, or permanent structures within the study period. The physical activity interventions implemented in this study did not require substantial investment or permanent changes to the built environment. Previous work in Appalachia indicates parks are generally available and accessible but connectivity infrastructure, such as sidewalks and crosswalks, walking trails, and biking trails are limited, influencing overall walkability of the region (59). Individuals may be willing to engage in these activities, but limited access to these resources continues to be a barrier to physical activity engagement within Appalachia (60). While features to improve activity levels may exist, there is an assumption made that physical activity habits will increase if larger changes to the environment could occur over a longer time frame. However, these environmental changes may not increase motivation of community residents, who share they have no desire to be physically active (40). In addition, several systemic challenges compound the dilapidated physical infrastructure that could contribute to low levels of physical activity among community members. These include social distancing and isolation from the COVID-19 pandemic, a population with high rates of physical disability, and the loss of physically demanding jobs in the region (e.g., coal mining).

The COVID-19 pandemic layered on the pervasive and persistent challenges related to physical infrastructure in the community challenge personal safety as well as economic impacts. These exogenous factors may have had a greater impact on daily choices relative to community improvements, such as marketing in gas stations. Research has highlighted how COVID-19 was related to food insufficiency and especially among rural underserved communities. Thus, our results may suggest how COVID-19 and subsequent economic impacts influenced higher rates of food sufficiency and thus the discrepancy in dietary intake patterns (61–63). Pressing issues like poor housing, inadequate food access, dilapidated water infrastructure, and limited disaster response are compounded even more by the geographic remoteness and mountainous terrain of the region. This may account for or partially explain the lack of change in dietary and physical activity habits observed in this cohort study.

While our findings are mixed in relation to the outcomes of interest, community organizations, public health entities, and Extension should not be dismayed from future work by the lack of positive impact on dietary and physical activity habits within this short timeframe. There are still broad implications from this project that could inform future interventions in rural communities that have limited infrastructure and resources to support healthy living. First, while there were only slight changes in dietary outcomes, there is a positive outlook for vegetable consumption. Other published findings from this community suggest there is broad support for community gardens and local food systems efforts that bring nutritious food into the community (38, 39, 51). There are opportunities to explore other interventions such as mobile food operations and bolster collaborations among local food banks and pantries. Second, these physical activity outcomes reflect the qualitative data that we collected in this community where activity was not identified as a priority (40). Increasing physical activity among the broader population will likely require local partnerships to integrate creative strategies into existing programs and to leverage local resources, where physical activity is not the promoted activity but rather a byproduct of the event or program. For example, the StoryWalk® installation could be showcased as a mechanism to bolster family bonding and early literacy (43), while subsequently increasing physical activity over time. Finally, Extension was an effective facilitator managing several community-driven projects simultaneously. The trusted and established relationships Extension has with numerous community partners is what allowed so many interventions to be implemented within a three-year period. The success of initiatives is only possible due to investment in relationships with key community partners and stakeholders, driven by the work of Extension, which has been previously demonstrated in rural communities (64, 65).

### 4.1. Limitations and considerations

This study’s data collection period spanned three years, which may not be long enough to see substantial changes in health-related habits or to see improved health outcomes at a population level. Often restricted by funding mechanisms, there is little evidence to suggest how long studies should be to see behavior change and subsequent population level health improvements following the implementation of PSE interventions (66). Further, these findings are limited in the generalizations that can be made to other rural communities because data were collected in one rural Appalachian County. Although validated tools were used to reduce bias, self-reported data may include social desirability or recall bias since the surveys were verbally administered. Trained study personnel administered surveys to reduce errors, missing data, and concerns of low literacy levels on survey completion. From baseline to follow-up, we experienced 38.8% attrition. However, similar levels of attrition have been reported for other community-based projects assessing health behavior change (67, 68). Finally, it should be noted that this research team is comprised of white women who work within a higher education setting and within the Extension system in a metro area. These factors introduce inherent bias when interpreting data and analyzing findings within the context of a different setting.

While not a limitation of the study design, the COVID-19 pandemic unfolded during the study period. Additionally, the county experienced substantial flooding requiring federal assistance and one of three local grocery stores permanently closed during the implementation of interventions and data collection. Further, the county was impacted by turnover in local elected leadership, with three county judge executives being elected or appointed over a three-year span. This change in leadership impacted or delayed progress for project interventions at various timepoints.

### 4.2. Conclusion

This study assessed whether community-level PSE interventions and programming implemented within the CDC HOP project resulted in individual health behavior change through a prospective cohort study in rural Appalachia. Using tailored PSEs to support health inherently addresses the inequities the community experiences and is an important piece needed for supporting health behavior change across a broader population. While this study was unable to move the needle substantially over a three-year period on diet and physical activity measures, there were promising trends and important considerations gleaned. These findings are applicable and relevant for all practitioners, public health professionals, and Extension personnel working within rural communities to address health behaviors and ultimately improve health outcomes.

## Data availability statement

The raw data supporting the conclusions of this article will be made available by the authors, without undue reservation.

## Ethics statement

The studies involving human participants were reviewed and approved by University of Kentucky Institutional Review Board (approved protocol #40895). The patients/participants provided their written informed consent to participate in this study.

## Author contributions

KC, ED, RG, and HN-B: conceptualization. KC and AG: methodology. SS, RG, and ED software. HN-B and AG: validation. SS: formal analysis and data curation. HN-B, ED, and RG: investigation. HN-B, ED, RG, and AG: resources. HN-B, ED, and RG: writing—original draft preparation. KC, SS, and AG: writing—review and editing. AG and KC: visualization. AG and KC: supervision. RG, ED, and HN-B: project administration. KC and AG: funding acquisition. All authors contributed to the article and approved the submitted version.

## Funding

This research was funded by Centers for Disease Control and Prevention (CDC) Division of Nutrition, Physical Activity, and Obesity (DNPAO), Cooperative Agreement number 1NU58DP0065690100.

## Conflict of interest

The authors declare no conflict of interest. The research was conducted in the absence of any commercial or financial relationships that could be construed as a potential conflict of interest.

## Publisher’s note

All claims expressed in this article are solely those of the authors and do not necessarily represent those of their affiliated organizations, or those of the publisher, the editors and the reviewers. Any product that may be evaluated in this article, or claim that may be made by its manufacturer, is not guaranteed or endorsed by the publisher.

## Footnotes

^1^ https://www.chfs.ky.gov/agencies/dph/dpqi/cdpb/dpcp/2021DiabetesReport.pdf

^2^ https://wellconnectedcommunities.org/about/

^3^ https://www.cdc.gov/nccdphp/dnpao/state-local-programs/hop-1809/hop-1809-recipients.html

^4^ https://www.census.gov/data-tools/demo/saipe/#/

^5^ https://www.cdc.gov/obesity/data/adult.html

^6^ https://www.census.gov/data-tools/demo/saipe_treemap/saipe_snap_treemap.html

^7^ https://www.rwjf.org/en/library/interactives/whereyouliveaffectshowlongyoulive.html

^8^ https://www.kellogghubbard.org/storywalk

^9^ https://epi.grants.cancer.gov/diet/screeners/fruitveg/scoring/allday.html
